# On the Powers of Quinine in Remittent Fevers

**Published:** 1850-05

**Authors:** 


*Remarks of Dr. Isaac Parrish, of Philadelphia, on the powers of Quinine in Remittent Fever.*

The following remarks by Dr. Parrish, were made at a meeting of the Philadelphia County Medical Society. They occasioned a discussion in which several members took part, some of whom dissented from the views expressed by Dr. P. We should give the latter, were they not too extended to be comprised in the space we can allot to the subject. We select Dr. Parrish's remarks, because they accord with the conclusions to which we have been led by our own observations, and which we have taught for several years. The subject possesses much practical importance, and we commend it to the attention of practitioners residing in miasmatic districts.

Our practice in cases of Remitting Fever, is to commence as early as practicable with the Quinine, not delaying even for the action of a "brisk mercurial cathartic." And we are satisfied that by a judicious use of this remedy, the progress of the febrile disease, in a large proportion of cases, may be arrested. We copy the article which follows, from the Medical Examiner.—Editor Buffalo Medical Journal.

Dr. Parrish remarked that, in the absence of the lecturer for the evening, he would ask the attention of the Society to a subject of much practical interest, in the hope of eliciting inquiry, and of promoting discussion.

It is well known that, within a few years past, the practice of administering quinine in the early stage of malarial fevers of the remittent type, has become quite prevalent in those sections of the United States where these fevers are most prevalent. The idea is now extensively entertained, that remittent and intermittent fevers are essentially the same disease, and that the absence of distinct intermissions in the former, should not prevent the administration of the remedy which is found so effectual in controlling the latter. Hence, the physicians who hold to this opinion, give quinine in large doses, and with a view to its anti-periodic qualities, in the remissions of a fever, for the purpose of preventing the exacerbations, which are known to occur at particular intervals. They do not wait until all febrile action has passed away, or until a crisis occurs in the natural course of the fever, on the fifth, seventh, or ninth day, or even later, but they commence at the outset with this potent remedy, just as they would do if no fever were present, as in the intermission of an intermittent. In both cases, they would, perhaps, generally precede the quinine by a brisk mercurial cathartic, and then attack it with quinine, in the interval of the paroxysms, with a view to cutting short the disease.

The results of this practice, as reported to us by many highly respectable physicians of the west and south, whose experience has been ample, is, that remittent fevers which, under the plan of treatment formerly in vogue, were accustomed to run a protracted course, and to assume a serious aspect, are brought to a rapid termination, or converted, after two or three paroxysms, into the intermittent type, and easily cured. They say, that so far from the quinine increasing the heat, thirst, and sensorial disturbance incident to a fever, that it allays these, calms the nervous system, and acts as a diaphoretic. Now, it is a point of great practical interest, to know how far these observations of our brethren in other sections, correspond with experience here ; and my object in introducing the subject at this time is, to ascertain this.

For myself I can state that, in common with most of those who hear me, I was educated in the doctrine, that in remittents, we must be very cautious of bark or quinine, while the least febrile excitement is present.

The idea entertained here, by the most eminent medical teachers, when I was a student, was, that in remittent, the febrile excitement must be reduced by the lancet and brisk purgation during the early stages, and that tonics were only to be given after the decline of the fever. Even my excellent father, who, as is well known by many here, was more favorable to the use of tonics and supporting treatment in fever, than most of his contemporaries, taught in his lectures, that bark and quinine were not to be given until the cleaning of the tongue, or the occurrence of critical sweats indicated the approach of convalescence ; except in cases where malignant symptoms were developed, or where unusual evidence of prostration existed,—and then the article was given more for its general tonic properties, than with any prospect of cutting short the fever.

Within the past few years, however, from the large amount of testimony to the value of quinine in the early stages of remittent which has reached us through the medical journals, I have been induced to pursue this course, and have reason to be well satisfied with it. I have found that quinine, given in the early stage of a remittent, after a free evacuation of the bowels by a mercurial cathartic, and when a distinct morning remission was observable, has had the effect of moderating the evening exacerbation, and, by continuing it a few days, of bringing the fever to a rapid and favorable crisis. I have seldom given over 15 or 20 grains in the remission, and in mild cases, have found even less than this to answer the purpose. Since adopting this plan I have not seen any cases of protracted remittent, running on for three and four weeks, and attended with tympanitis, subsultus tendinum, low delirium, hæmorrhage from the bowels, &c. Whether this fortunate exemption is attributable to the mode of treatment adopted, or to a diminished liability in our fevers of late years to assume this form, I cannot positively say; though I am inclined to think that the old plan of delaying the use of tonics, may have contributed to the production of these prolonged cases.

If the practice recently introduced by the practitioners of the south and west, in the treatment of malarial fevers, be confirmed by experience, and established as sound,it must be admitted that a great step has been taken in practical medicine; diseases, once the dread of the inhabitants of large sections of our country, will be divested of much of their virulence, and be brought under the control of remedial measures, while our science will have achieved a grand triumph.

